# Evaluation of an ambulatory system for the quantification of cough frequency in patients with chronic obstructive pulmonary disease

**DOI:** 10.1186/1745-9974-1-3

**Published:** 2005-08-04

**Authors:** Michael A Coyle, Desmond B Keenan, Linda S Henderson, Michael L Watkins, Brett K Haumann, David W Mayleben, Michael G Wilson

**Affiliations:** 1 Physiology Program, Harvard School of Public Health, Boston, MA, USA; 2 VivoMetrics, Inc., Ventura, CA, USA; 3 GlaxoSmithKline, Respiratory and Inflammation Centre of Excellence for Drug Discovery Research Triangle Park, NC, USA; 4 GlaxoSmithKline, Respiratory and Inflammation Centre of Excellence for Drug Discovery Stevenage, UK; 5 Community Research, Inc., Cincinnati, OH, USA; 6 Department of Psychiatry, Indiana University School of Medicine, Indianapolis, IN, USA

## Abstract

**Background:**

To date, methods used to assess cough have been primarily subjective, and only broadly reflect the impact of chronic cough and/or chronic cough therapies on quality of life. Objective assessment of cough has been attempted, but early techniques were neither ambulatory nor feasible for long-term data collection. We evaluated a novel ambulatory cardio-respiratory monitoring system with an integrated unidirectional, contact microphone, and report here the results from a study of patients with COPD who were videotaped in a quasi-controlled environment for 24 continuous hours while wearing the ambulatory system.

**Methods:**

Eight patients with a documented history of COPD with ten or more years of smoking (6 women; age 57.4 ± 11.8 yrs.; percent predicted FEV_1 _49.6 ± 13.7%) who complained of cough were evaluated in a clinical research unit equipped with video and sound recording capabilities. All patients wore the LifeShirt^® ^system (LS) while undergoing simultaneous video (with sound) surveillance. Video data were visually inspected and annotated to indicate all cough events. Raw physiologic data records were visually inspected by technicians who remained blinded to the video data. Cough events from LS were analyzed quantitatively with a specialized software algorithm to identify cough. The output of the software algorithm was compared to video records on a per event basis in order to determine the validity of the LS system to detect cough in COPD patients.

**Results:**

Video surveillance identified a total of 3,645 coughs, while LS identified 3,363 coughs during the same period. The median cough rate per patient was 21.3 coughs·hr^-1 ^(Range: 10.1 cghs·hr^-1 ^– 59.9 cghs·hr^-1^). The overall accuracy of the LS system was 99.0%. Overall sensitivity and specificity of LS, when compared to video surveillance, were 0.781 and 0.996, respectively, while positive- and negative-predictive values were 0.846 and 0.994. There was very good agreement between the LS system and video (kappa = 0.807).

**Conclusion:**

The LS system demonstrated a high level of accuracy and agreement when compared to video surveillance for the measurement of cough in patients with COPD.

## Background

The most frequent complaint for which patients seek treatment from primary care physicians in the United States is cough [1]. Type, frequency and diurnal changes of cough may be criteria for differential diagnosis, therapeutic efficacy, and a gauge for the progression of chronic disease. Historically, cough evaluation has been difficult and of limited clinical value due to a lack of surveillance tools to assess cough frequency completely and its impact on health-related quality of life (HRQL).

To date, methods used to assess cough have been primarily subjective, and only broadly reflect the impact of chronic cough and/or chronic cough therapies on quality of life [2-5]. These methods have been unable to offer substantial information related to the minimal reduction in cough frequency necessary to achieve a significant improvement in HQRL. Objective assessment of cough has been attempted, but these techniques were neither ambulatory nor feasible for long-term data collection [6-8]. Other systems have evaluated sound to quantify cough frequency and intensity with moderate success [9,10], but have been limited in their effectiveness outside the laboratory and requires labor intensive analysis and interpretation [11-15].

We evaluated a novel ambulatory cardio-respiratory monitoring system with an integrated unidirectional, contact microphone, and report here the results from a study of patients with COPD who were videotaped in a quasi-controlled environment for 24 continuous hours while wearing the ambulatory system.

## Methods

### Subjects

Eight subjects with chronic obstructive pulmonary disease (COPD) who complained of cough as a prominent symptom (e.g., ten or greater self reported bouts of cough per day) were recruited for the study. Subjects were men and women over the age of 40 who had a documented medical history of COPD and a smoking history of ≥ 10 years with chronic productive cough. Patient characteristics can be found in Table 1. Patients were excluded from the study if, upon screening, (1) it was determined from patient medical history that cough could be due to other known causes such as gastro-esophageal reflux, asthma, or any anatomical abnormalities of the upper respiratory tract, and/or (2) if patients were using prescribed or over the counter anti-tussive medications within 24-hours of the start of the study.

**Table 1:** Patient characteristics. Values are means ± SD; Ht = height; Wt = weight; BMI = Body mass index; %FEV_1 _= % predicted forced expiratory volume in one second; n = 8 (6 women)

Variable	
Age (yrs)	57.4 ± 11.8
Ht (cm)	165.4 ± 7.2
Wt (kg)	76.1 ± 14.4
BMI (kg/m^2^)	27.8 ± 4.7
%FEV_1_	49.6 ± 13.7

The protocol was approved by an independent ethical review board (Western IRB, 3535 7th Avenue SW, Olympia, WA, USA, 98502) and all patients received a verbal and written description of the study and gave informed consent prior to participation. All data were collected under the medical supervision of board certified pulmonologists.

### Instrumentation and monitoring

#### LifeShirt^® ^System

Patients were fitted with the wearable LifeShirt^® ^system (LS, VivoMetrics, Inc., Ventura, CA, USA), which incorporates respiratory inductance plethysmography (RIP) for the non-invasive measurement of volume and timing ventilatory variables and has been described elsewhere [15-22]. The system also incorporates a unidirectional contact microphone, a single channel ECG, and a centrally located, 3-axis accelerometer. Data were processed and stored on a compact flash card that was housed within the recorder unit. Patients were invited to wear the LS system for a maximum of 24 hours.

### Video surveillance

Patients spent the testing period in an assigned room where the video monitoring equipment was installed. Patients were monitored via video recorder camera (low-lux) with unidirectional free-air microphone for the duration of the testing period. The video data stream was synchronized to the LS data stream by the coordination of the device clocks. The LS recorder has an on-board electronic diary which creates an event time stamp in the LS software data stream which was referenced to the video data time display to determine the beginning of the recording period. Patients were allowed free range of the research facility and were permitted to watch television, use the telephone, dine, take breaks and sleep.

### Data analysis and statistics

Raw physiologic data records were uploaded to a centralized data center and were visually inspected for quality by technicians. 94.1% of the data were interpretable and available for comparison to video. Specialized software (VivoLogic^®^, VivoMetrics, Inc., Ventura, CA, USA) was used to view the LS data and a proprietary algorithm housed within the software was used to identify cough from the physiologic recordings. LS data were visually inspected by two independent reviewers who remained blinded to the video data. Each noted the time (hour, minute and second) of each cough. These data were captured into a spreadsheet. Cough events (hour, minute, second, millisecond) identified by the LS software were exported into a separate spreadsheet. A practical extraction and report language (PERL) script was written to temporally align the two data streams so that the output from each device could be compared for agreement on an event by event basis.

To summarize the validity and reliability of the ambulatory system to detect cough under several conditions, six validation and agreement measures were used including, sensitivity (SN), specificity (SP), positive predictive value (PPV), negative predictive value (NPV), accuracy and kappa [23] were calculated relative to video rating. The PPV is the probability that a patient coughed, if the system judged the respiratory event as a cough. Likewise, the NPV is the probability that the patient did not cough, given that the system did not judge the event as a cough. Accuracy is the proportion of all correct tests. The method used to calculate the confidence intervals was the Wilson score method without continuity correction [24], which has been previously shown to exhibit a logit scale symmetry property with consequent log scale symmetry for certain derived intervals [25].

## Results

**Figure 1:**
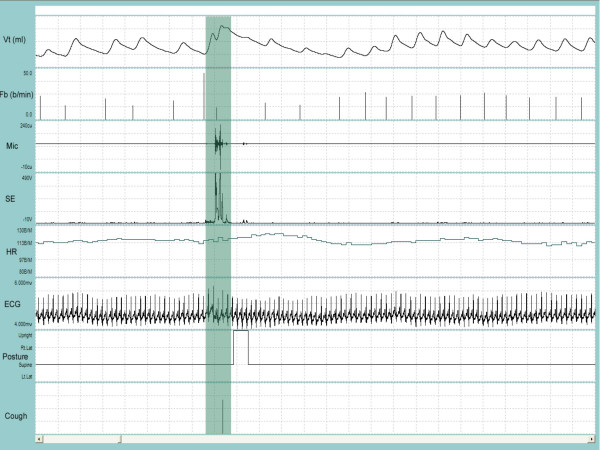
**Representative recording of a single cough followed by a throat clear during quiet breathing**. V_T _= tidal volume; F_b _= breathing frequency; Mic = contact microphone output; SE = sound envelope; HR = heart rate; ECG = electrocardiograph tracing; Posture = body position defined as upright, supine, right decubitis and left decubitis; Cough = cough output from algorithm. The shaded bar contains the cough event. Cough is indicated by a single solid line at the end of the breath that contains the cough. Note the change in the posture from supine to upright to supine immediately following the cough. Entire duration of depicted recording is 1-min and 1-sec.

**Figure 2:**
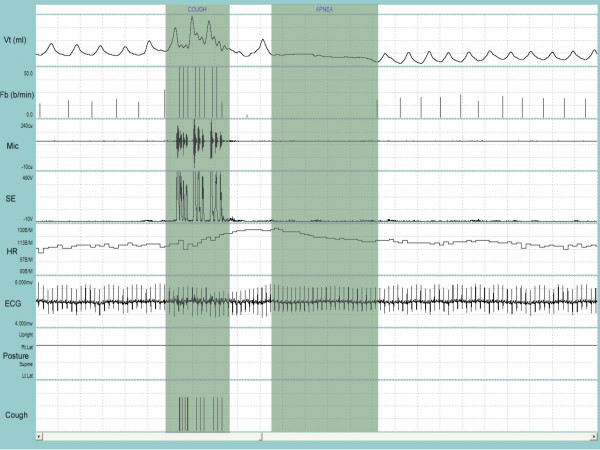
**Representative recording of coughing during sleep**. V_T _= tidal volume; F_b _= breathing frequency; Mic = contact microphone output; SE = sound envelope; HR = heart rate; ECG = electrocardiograph tracing; Posture = body position defined as upright, supine, right decubitis and left decubitis; Cough = cough output from LS algorithm. The first shaded bar contains the cough bout. Ten coughs occurred during the 9-sec bout. Each cough is indicated by a single solid line at the end of the breath that contains the cough. Note that the cough bout was followed by a 15-sec apnea (second shaded bar). Entire duration of depicted recording is 1-min and 23-sec.

**Figure 3:**
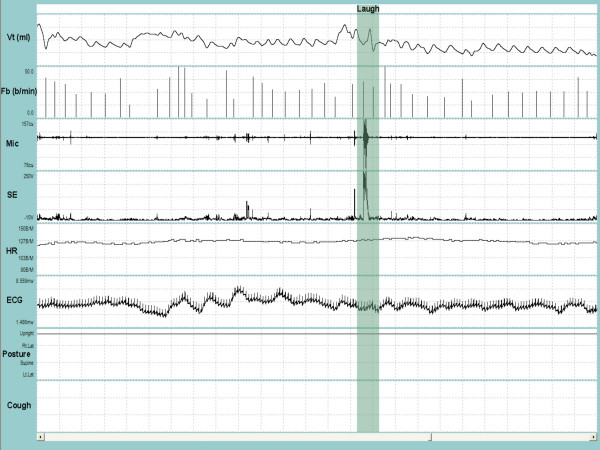
**Representative recording of talking and laughing**. V_T _= tidal volume; F_b _= breathing frequency; Mic = contact microphone output; SE = sound envelope; HR = heart rate; ECG = electrocardiograph tracing; Posture = upright. The shaded bar contains a burst of laughing. Entire duration of depicted recording is 1-min and 37-sec.

A satisfactory fit of the available standard sizes of the respiratory inductance plethysmography (RIP) garment was achieved in all patients and the system was well tolerated during the recording period. Figure 1 and Figure 2 depict a representative recording of a single cough during quiet breathing and during a series of coughs close together, respectively. Figure 3 is a representative recording of speech.

Patients were invited to be observed for a maximum of 24 hours. A total of 109 hours of simultaneous recordings of video and LS were obtained. Of that time, 73.9 hours were observed during the day and 34.7 hours were observed during the night. During the recording period, the total number of coughs documented by video surveillance was 3,645. The LS system reported 3,363 coughs during the same time period. The median cough rate per patient was 21.3 coughs·hr^-1 ^(Range: 10.1 cghs·hr^-1 ^– 59.9 cghs·hr^-1^).

Table 2 provides performance summaries for the LS system to detect cough during for night vs. day and for low and high respiration rates, respectively. Patients were assigned to low vs. high respiratory rate depending on whether the rate was below or above the median breathing frequency (median F_b _= 21 br·min^-1^). The system was highly accurate in identifying cough as a respiratory event during night or day. Accuracy during the night was 99.4%, while accuracy during the day was 98.8% for a difference = 0.53%. The specificities and negative predictive values are considered 'excellent' by the criteria proposed by Byrt (1996)[26]. Sensitivities, positive predictive values and kappa can be considered 'very good' by the same criteria. Likewise, the performance summaries for the system between high or low respiration rates were remarkably similar. Accuracy, specificities, and negative predictive values were 'excellent' and sensitivities, positive predictive values and kappa were 'very good' [26].

**Table 2:** Validation and agreement statistics (with 95% confidence intervals) for the LifeShirt system during day & night and at low & high respiration rates. Values are calculated values for the sensitivity (SN), specificity (SP), positive predictive-value (PPV), negative predictive-value (NPV), accuracy (ACC) and the kappa statistic. Values in parentheses are the 95% confidence intervals. All values are for LS system compared to video documentation of cough events; * p-value < 0.0001 for night vs. day comparisons of period for SN, SP, NPV and ACC; ¶p-value < 0.0001 for night vs. day comparisons of respiratory rate for SN, SP, PPV, NPV; ‡ p-value = 0.004 for day vs. night comparisons of respiratory rate for ACC. Day period defined as 0600–1800; Night period defined as 1800–0600. Patients were assigned to the low or high respiratory rate group based on whether their mean F_b _was below or above the median F_b _(median = 21 br·min^-1^).

		**Period**		**Respiratory Rate**	
			
	**Combined**	**Day**	**Night**	**Low**	**High**
**SN**	78.1 (76.7, 79.4)	76.7 (75.1, 78.2)	82.7 (80.0, 85.1)*	69.5 (66.3, 72.6)	80.6 (79.0, 82.0)¶
**SP**	99.6 (99.5, 99.6)	99.6 (99.5, 99.6)	99.7 (99.7, 99.8)*	99.5 (99.5, 99.6)	99.7 (99.6, 99.7) ¶
**PPV**	84.6 (83.3, 85.8)	84.5 (83.0, 85.8)	85.0 (82.3, 87.3)	69.8 (66.5, 72.8)	89.4 (88.1, 90.5) ¶
**NPV**	99.4 (99.3, 99.4)	99.3 (99.2, 99.3)	99.7 (99.6, 99.7)*	99.5 (99.5, 99.6)	99.3 (99.2, 99.3) ¶
**ACC**	99.0 (99.0, 99.1)	98.8 (98.8, 98.9)	99.4 (99.3, 99.5)*	99.1 (99.0, 99.2)	99.0 (98.9, 99.0)‡
**Kappa**	80.7 (79.7, 81.7)	79.8 (78.6, 81.0)	83.5 (81.5, 85.5)	69.2 (66.6, 71.7)	84.2 (83.1, 85.3)

## Discussion

We report validity and reliability statistics for a novel ambulatory system to evaluate its capability to detect cough and demonstrate a high level of agreement and accuracy when compared to video surveillance for cough over an extended period. The system was well-tolerated and allowed for free movement throughout the monitoring facility. The system continuously monitors several cardio-pulmonary-activity variables, which allowed us to evaluate ventilatory strategies associated with coughing, which is one of its novel features.

The patient population in this study coughed with great frequency, which reflects the fact these were COPD patients who had a primary complaint of cough. At screening, patients were asked if they coughed ten times per day or more. Although all of the patients met this requirement, they had difficulty recalling how many cough bouts per day they experienced. As such, we did not predict that this population would cough with such a high frequency and, although the number of coughs was higher than anticipated, it was within the range of what has been reported previously [12].

Agreement between the LS system and video surveillance was excellent. Interestingly, we did observe that the nocturnal validation and agreement statistics, as well as differences between low and high respiratory rates, were statistically significantly different, although they differed only slightly in magnitude. These small, yet statistically significant, difference likely reflect the influence of the respiratory events (e.g., V_T _and F_b_) sample size during the recording period on the statistical power for these comparisons and is not clinically significant.

Objective cough assessment has been attempted on numerous previous occasions [6-8,10,12,14]. Until now, a robust, accurate ambulatory system has failed to emerge. This is likely due to the fact that previous systems have attempted to identify cough with a single physiologic signal (e.g., sound). Sound-based technologies have been the primary means of cough assessment due to the audible sound that is generated during a cough [28]. These systems, however, are susceptible to a high false positive rate when ambient noise is prominent and are unable to distinguish cough-like sounds (e.g., throat clearing) from true cough. Hsu et al. (1994) [12] augmented sound analyses with concomitant intercostal electromyography (EMG) analyses and evaluated various clinical populations (e.g., normal controls, stable asthmatics and patients with daily, persistent & non-productive cough) and concluded that their system may be useful in the assessment of antitussive therapies. Hsu et al., however, did not present evidence of agreement by comparing their results to a reference standard.

There were three limitations to the study. First, the sample size was small. Sample size was limited by available resources to review the vast amount of video and LS data. Second, women (6/8) were over represented in the study, which was due to an inauspicious baseline imbalance. Third, the data were collected in a clinical setting due to the requirement for video monitoring equipment. However, patients were not confined to any one space and ambulated, spoke on the phone, dined and performed additional activities of daily living.

A substantial challenge in this study was the choice of a reference standard with which to evaluate the novel device. We chose video based on the fact that the source document (video) could be reviewed during the adjudication process if there was uncertainty with respect to the occurrence of a cough. Scoring the video in duplicate was an arduous task which likely increased the possibility of human error due to fatigue and it is possible that some coughs were missed. Events that were missed by both reviewers would not have been adjudicated, but identified by the LS system and therefore would have been inappropriately scored as a false-positive. Thus, these results are a conservative estimate of LS capabilities and may underestimate the predictive power of the device.

## Conclusion

We report data from a novel, ambulatory, multi-signal device that shows a high level of agreement and accuracy when compared to video/audio surveillance over an extended period and confirm its potential in the evaluation of antitussive therapies. The availability of a valid, robust ambulatory tool for quantifying cough will enable the determination of the minimal required reduction in cough to maximally improve patient HQRL, and open up a broad array of research questions both specific to cough and wherein cough may be an important covariate, comorbidity, or confounding factor.

## Competing interests

Financial Disclosure: MAC and DBK were employed by VivoMetrics during the course of the study. MGW consults for VivoMetrics. LSH, MLW, BKH are employed by GlaxoSmithKline. Supported by a grant from GlaxoSmithKline.

## References

[B1] Schappert SM (1993). National ambulatory medical care survey: summary. Vital Health Stat.

[B2] Birring SS, Prudon B, Carr AJ, Singh SJ, Morgan MD, Pavord ID (2003). Development of a symptom specific health status measure for patients with chronic cough: Leicester Cough Questionnaire (LCQ). Thorax.

[B3] French CL, Irwin RS, Curley FJ, Krikorian CJ (1998). Impact of chronic cough on quality of life. Arch Intern Med.

[B4] Irwin RS, French CT, Fletcher KE (2002). Quality of life in coughers. Pulm Pharmacol Ther.

[B5] French CT, Irwin RS, Fletcher KE, Adams TM (2002). Evaluation of a cough-specific quality-of-life questionnaire. Chest.

[B6] Barach AL, Bickerman HA, Beck GJ (1955). Clinical and physiological studies on the use of metacortandracin in respiratory disease.1.Bronchial asthma. DC.

[B7] Bickerman HA, Barach AL (1954). The experimental production of cough in human subjects induced by citric acid aerosols. Peliminary studies in the evaluation of antitussive agents. Am J Med Sci.

[B8] Prime FJ (1961). The assessment of antitusive drugs in man. Br Med J.

[B9] Woolf CR, Rosenberg A (1962). The cough suppressant effect of heroin and codeine: a controlled clinical study. Can Med Assoc J.

[B10] Sevelius H, Colmore JP (1966). Objective assessment of antitussive agents in patients with chronic cough. J New Drugs.

[B11] Chang AB, Phelan PD, Robertson CF, Newman RG, Sawyer SM (2001). Frequency and perception of cough severity. J Paediatr Child Health.

[B12] Hsu JY, Stone RA, Logan-Sinclair RB, Worsdell M, Busst CM, Chung KF (1994). Coughing frequency in patients with persistent cough: assessment using a 24 hour ambulatory recorder. Eur Respir J.

[B13] Dalmasso F, Isnardi E, Sudaro L, Bellantoni R (2001). Bioacoustins of cough during bronchial inhalation challenge (BIC) with methacholine. Eur Resp J.

[B14] Subburaj S, Parvez L, Rajagopalan TG (1996). Methods of recording and analysing cough sounds. Pulm Pharmacol.

[B15] Tobin MJ, Chadha TS, Jenouri G, Birch SJ, Gazeroglu HB, Sackner MA (1983). Breathing patterns. 1. Normal subjects. Chest.

[B16] Tobin MJ, Chadha TS, Jenouri G, Birch SJ, Gazeroglu HB, Sackner MA (1983). Breathing patterns. 2. Diseased subjects. Chest.

[B17] Chadha TS, Schneider AW, Birch S, Jenouri G, Sackner MA (1984). Breathing pattern during induced bronchoconstriction. Journal of Applied Physiology: Respiratory, Environmental & Exercise Physiology.

[B18] Sackner MA, Gonzalez HF, Jenouri G, Rodriguez M (1984). Effects of abdominal and thoracic breathing on breathing pattern components in normal subjects and in patients with chronic obstructive pulmonary disease. American Review of Respiratory Disease.

[B19] Tobin MJ, Guenther SM, Perez W, Mador MJ (1987). Accuracy of the respiratory inductive plethysmograph during loaded breathing. Journal of Applied Physiology.

[B20] Cantineau JP, Escourrou P, Sartene R, Gaultier C, Goldman M (1992). Accuracy of respiratory inductive plethysmography during wakefulness and sleep in patients with obstructive sleep apnea. Chest.

[B21] Carter GS, Coyle MA, Mendelson WB (2004). Validity of a portable cardio-respiratory system to collect data in the home environment in patients with obstructive sleep apnea. Sleep and Hypnosis.

[B22] Clarenbach CF, Senn O, Brack T, Bloch KE (2005). Monitoring of ventilation during exercise by a novel portable respiratory inductive plethysmograph. Chest, In Print.

[B23] Greenhalgh T (1997). How to read a paper. Papers that report diagnostic or screening tests. BMJ.

[B24] Wilson EB (1927). Probable inference, the law of succession, and statistical inference. J Am Stat Assoc.

[B25] Newcombe RG (1998). Two-sided confidence intervals for the single proportion: comparison of seven methods. Stat Med.

[B26] Byrt T (1996). How good is that agreement? (Letter to editor). Epidemiology.

